# Looking Beyond Thrombocytopenia for Bleeding in a Case of Dengue Fever: From Dengue to Hemophilia C

**DOI:** 10.7759/cureus.72688

**Published:** 2024-10-30

**Authors:** Shweta S Acharya, Praveen Arumugam, Ashok Kumar, Pankhuri Kumari, Ashok Kumar

**Affiliations:** 1 Internal Medicine, Max Smart Super Speciality Hospital, New Delhi, IND; 2 Microbiology, Max Smart Super Speciality Hospital, New Delhi, IND; 3 Microbiology, Amrita Hospital Faridabad, Faridabad, IND

**Keywords:** coagulopathy, dengue fever, factor xi deficiency, gastrointestinal bleed, haemophilia c, thrombocytopenia

## Abstract

Dengue fever causes thrombocytopenia, which can present with bleeding manifestations. Bleeding can range from less severe conditions like petechiae, epistaxis, and menorrhagia, to life-threatening situations like gastrointestinal bleed or intracranial bleed. We present a case of a young boy in his early twenties, who was admitted with Dengue fever, presenting with malena and blood in vomitus. He was managed on the lines of severe dengue with bleeding. However, malena, with a concurrent drop in haemoglobin, persisted despite normalisation of the platelet count. He was evaluated further and was found to have coagulopathy with factor XI deficiency (Hemophilia C) and a bleeding gastroesophageal junction ulcer.

## Introduction

Thrombocytopenia in dengue fever is a known cause for bleeding. It may lead to superficial minor bleed to even major organ bleed. In certain cases with underlying coagulopathy with factor deficiency, bleeding initiated by thrombocytopenia due to dengue fever can be prolonged. In this case, a young boy in his early 20s with dengue fever had upper gastrointestinal bleed which persisted even after normalization of the platelet counts. This raised a suspicion to further evaluate for the cause of prolonged bleeding and he was found to have underlying hemophilia C. A similar case was reported by Wijayaratne et al. wherein a young male had dengue fever with bleeding manifestation, which was worsened with concurrent factor VIII deficiency, later treated with factor VIII infusion [1]. Alike our patient's coagulopathy and bleeding manifestations were also improved on administering fresh frozen plasma and had no further symptoms and drop in hemoglobin on follow-up.

## Case presentation

A young male in his early 20s presented with a history of fever with chills for five days along with generalised weakness and body ache. In addition, he had nausea and vomiting for the past five days, non-bilious and containing food particles. He had one episode of blood in vomitus two days prior to the admission, along with complaints of black stools for the past five days. He denied any history of breathlessness, cough, burning micturition, loose stools, rash or joint pains. His blood reports on day 4 of fever showed dengue NS1 antigen positive and a platelet count of 49 x 10^9^/L. He did not have any previous medical conditions or any history of bleeding in the past. On examination, he was febrile and tachycardic with a blood pressure of 100/70 mmHg.

Abdominal examination revealed mild epigastric tenderness. The patient appeared dehydrated and was started on IV fluid bolus followed by maintenance along with Proton-pump Inhibitor intravenous infusion. Furthermore, the patient had 2-3 episodes of black stools and became lethargic. His hemoglobin (Hb) dropped from 11.7 to 8.9 gm/dL (Table 1), and other lab reports showed raised aspartate aminotransferase (AST), alanine aminotransferase (ALT) and aPTT (activated partial thromboplastin time). His PT (prothrombin time) and INR (international normalized ratio) were within acceptable limits. He was thus shifted to a medical intensive care unit for further management. His hemoglobin showed a further drop to 6.4 gm/dL. A mixing study for aPTT was done, which revealed partial correction; thus samples were sent for factor assay and factor inhibitors.

**Table 1:** List of investigations done at the time of hospitalisation. USG: Ultrasonography, UGI: Upper gastrointestinal

Investigations	Patient’s value	Normal range with units
Hemoglobin (Hb)	8.9 g/dL	13-17 g/dL
Total Leucocyte Count (TLC)	2.88 x 10^9^/L	4.0-10.0 x 10^9^/L
Platelet Count	125 x 10^9^/L	150-400 x 10^9^/L
Prothrombin Time (PT)	12.8 sec	10.2-13.6 sec
International Normalized Ratio (INR)	1.1	
Aspartate aminotransferase (AST)	376 IU/L	15-41 IU/L
Alanine aminotransferase (ALT)	152 IU/L	17-63 IU/L
Albumin	3.6 g/dL	3.5-5.0 g/dL
aPTT (activated partial thromboplastin time)	70.7 sec	24.7-34.8 sec
aPTT post mixing study	38 sec	sec
Fibrinogen	257 mg/dL	238-498 mg/dL
Fibrinogen degradation product (FDP)	5-20 ug/dL	0-5 ug/dL
Serum Creatinine	0.7 mg/dL	0.5-1.04 mg/dL
Serum Sodium	138 mmol/L	136-144 mmol/L
Serum Potassium	4.28 mmol/L	3.6-5.1 mmol/L
Stool for occult blood	Positive	
Beta 2 microglobulin IgM	2.9 U/mL	<7 (Negative) U/mL
Beta 2 microglobulin IgG	1.2 U/mL	<7 (Negative) U/mL
Anti-phospholipid antibody IgM	2.47 U/mL	<10 (Negative) U/mL
Anti-phospholipid antibody IgG	1.01 U/mL	<10 (Negative) U/mL
Anti-Cardiolipin Antibody IgM	2.4 U/mL	<10 (Negative) U/mL
Anti-Cardiolipin Antibody IgG	2.2 U/mL	<10 (Negative) U/mL
Anti-nuclear antibody (ANA)- immunofluorescence	Negative	
Factor XI functional assay	23.5 U/dL	65-150 U/dL
Paired blood culture	Burkholderia cepacia	
USG whole abdomen	Gallbladder wall edema with mild ascites. Normal liver echotexture.	
UGI endoscopy	Bleeding ulcer at gastro-oesophageal junction	
Chest X-ray	No focal lesion seen in the lung parenchyma	

**Figure 1:**
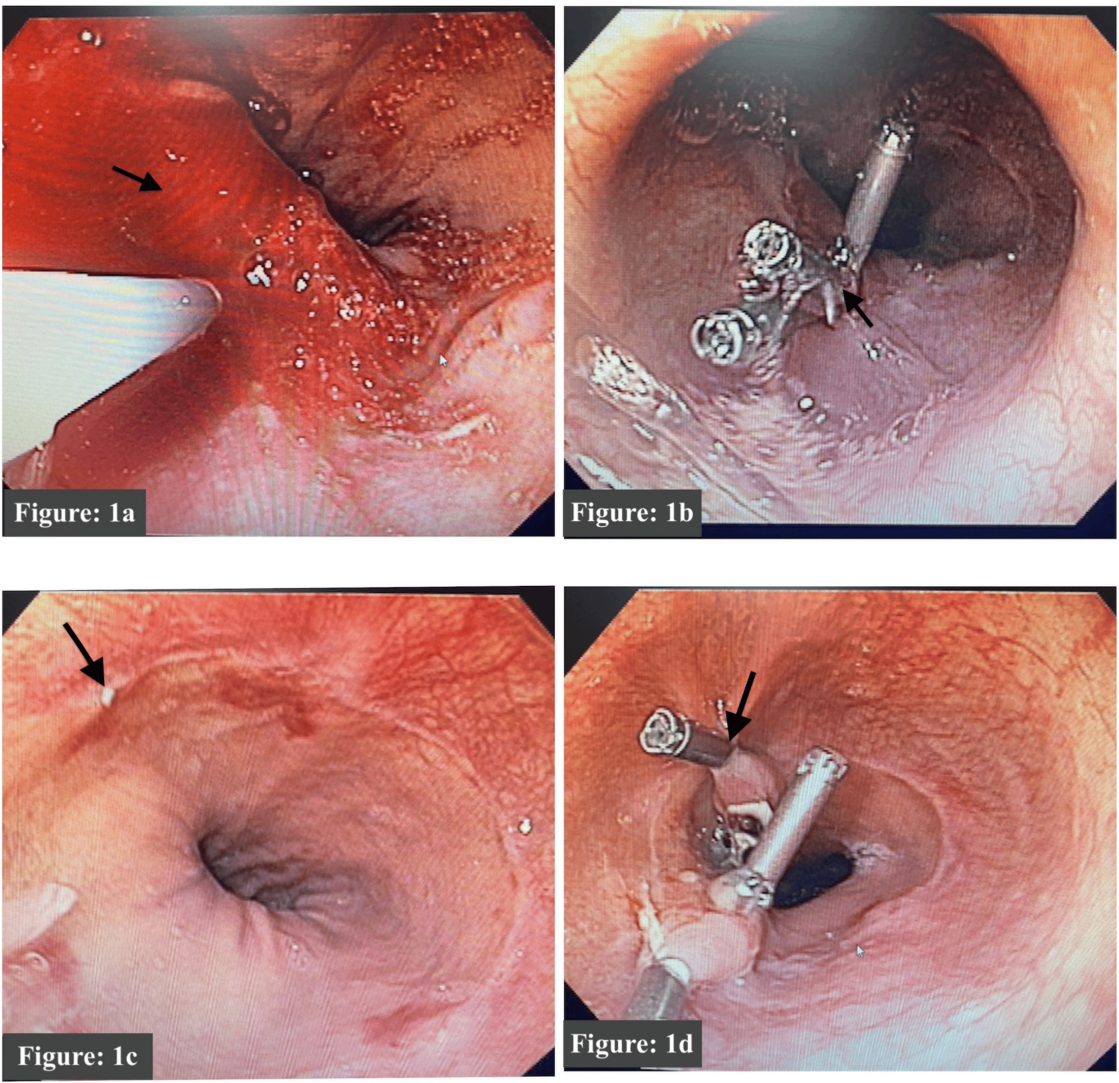
(a) UGIE image showing oozing of fresh blood seen at gastro-esophageal junction (black arrow). (b) Hemoclips (black arrow) were applied at the site of bleeding, and hemostasis was achieved. (c) Check upper gastrointestinal endoscopy done two days later; hemoclip applications showed areas of erosion (black arrow). (d) During check upper gastrointestinal endoscopy, another hemoclip was applied to achieve complete hemostasis (marked with a black arrow). UGIE: Upper Gastro-Intestinal Endoscopy

A gastroenterology consultation was taken because of malena and an upper gastrointestinal endoscopy was done, which showed a gastroesophageal junction ulcer (Figure 1a, 1b) with active bleeding. Thus, hemoclips were applied, and hemostasis was achieved. He was transfused with two units of LDPRBC (leucocyte-depleted packed red blood cells) and four units of FFP (fresh frozen plasma) on the same day. Although his platelet counts showed an improving trend to 164 x 10^9^/L and ALT and AST showed an improving trend, his malena persisted, and Hb did not improve thus, a check upper gastrointestinal endoscopy was done, which showed erosions with ooze at the previous ulcer site where the hemoclip was applied (Figure 1b, 1c). In due course, in the hospital, a total of 5 units of LDPRBC and 12 units of FFP were transfused.

Meanwhile, his paired blood culture showed *Burkholderia cepacia*, for which he was started on Inj. Levofloxacin 750mg once daily intravenously, as per the sensitivity report. On obtaining further detailed history, the patient's mother gave a history of factor XI deficiency, which was diagnosed during her pregnancy, as she had postpartum bleeding and required cryoprecipitate transfusion. Given the strong family history, a Factor assay was sent, which showed low levels of Factor XI. At the same time, the inhibitory markers such as Lupus anti-coagulation, anti-phospholipid antibody and beta-2 microglobulin were negative. Anti-nuclear antibody was also done and was reported negative. Thus, a diagnosis of upper gastrointestinal bleed due to factor XI deficiency was made, which was precipitated by the thrombocytopenia as well as coagulopathy induced by dengue fever. Upon administering the adequate amount of fresh frozen plasma, our patient's coagulopathy and bleeding manifestation improved. The patient was discharged once the hemoglobin was stable. On follow-up after seven days, the patient had no further complaints of malena and drop in hemoglobin.

## Discussion

Apart from thrombocytopenia leading to bleeding, dengue fever leads to an increased risk of coagulopathy, which can precipitate any underlying coagulation factor deficiency. Coagulopathy in dengue occurs due to several mechanisms as follows:

Platelet dysfunction and thrombocytopenia

Dengue virus infection leads to decreased platelet production and increased platelet destruction. Platelet counts fall during the febrile stage and reach their lowest during the critical phase [2]. As per Funahara et al. this is due to bone marrow suppression, increased platelet destruction, and consumption from interactions with dengue-infected endothelial cells [3].

Endothelial dysfunction

The virus and immune response cause endothelial cell activation and damage, leading to increased vascular permeability and plasma leakage [4]. This contributes to hemoconcentration and shock [4,5].

Activation of coagulation and fibrinolysis

There is activation of both coagulation and fibrinolytic systems. Coagulation factors such as prothrombin, V, VII, VIII, IX, and X are reduced, and there is an increase in tissue factor (TF) and thrombin-antithrombin complex (TAT) [6]. Fibrinolytic activity is also altered, with increased tissue-plasminogen activator (t-PA) and plasminogen activator inhibitor-1 (PAI-1) and decreased thrombin activatable fibrinolysis inhibitor (TAFI).

Consumption of Natural Anticoagulants

Levels of protein C and protein S are reduced, especially in severe cases, due to consumption and liver damage [7].

Disseminated intravascular coagulation (DIC)

In severe cases, especially those with shock, DIC can occur, characterised by widespread clotting and bleeding as mentioned in an article by Chuansumrit and Chaiyaratana. These mechanisms collectively lead to coagulopathy, contributing to the bleeding tendencies and complications seen in dengue hemorrhagic fever (DHF) and dengue shock syndrome (DSS) [5].

In this patient, the platelet count has improved to 125 x 10^9^/dL, thus, thrombocytopenia as a cause of upper gastrointestinal bleed was ruled out. Furthermore, other causes of thrombocytopenia and bleeding like hemolytic uremic syndrome (HUS) and thrombotic thrombocytopenic purpura (TTP) were ruled out given the absence of renal function derangement, absence of microangiopathic hemolytic anaemia and recovered thrombocytopenia. Normal liver echotexture and absence of liver surface nodularity on ultrasonography of the abdomen, along with normal serum albumin, ruled out chronic liver disease as a cause of coagulopathy. Moreover, raised aPTT and normal PT, INR, improved platelet count, no evidence of bleeding from puncture sites and hemodynamic stability along with normal fibrinogen levels ruled out disseminated intravascular coagulation (DIC). Taking into consideration the partial correction of aPTT in Mixing studies, suspicion of factor inhibitors was raised. The same has been ruled out in the light of negative tests for Lupus anticoagulant, anti-phospholipid antibody and beta 2 microglobulin. The test done for anti-nuclear antibody was also negative. Since the patient's aPTT improved after FFP and no further drop in hemoglobin occurred, along with the family history of factor XI deficiency in the mother and the presentation of deeper organ bleed, factor assay was sent, and the level was found to be low (23.5 U/dL). It was evident that the initial bleed might have occurred in an ulcer pre-existing at the gastroesophageal junction due to thrombocytopenia, which continued to bleed even after correction of platelet count as a part of delayed bleeding due to factor XI deficiency. Coagulopathy due to factor XI deficiency was suspected to be precipitated by coagulopathy occurring in Dengue fever.

In a similar case report from a teaching hospital in Sri Lanka, a 16-year-old boy having dengue fever presented with bleeding from the tooth extraction site and vomiting of dark red blood on day 3 of fever with a platelet count of 124 x 10^9^/L was later found to have factor VIII deficiency which responded to factor VIII transfusion as per Wijayaratne et al. [1]. Another study done by Chuansumrit et al. showed “increased bleeding risk during the early febrile stage of dengue fever with vasculopathy and continued to the late febrile stage in pediatric patients subgroup with underlying haemophilia, von Willebrand disease and thrombocytopenia" [8].

Out of the well-known congenital coagulation factor deficiencies, the severity of the symptoms may not correlate with the factor levels [9]. Severe deficiency is defined as factor XI activity of 15-20 U/dL or lower. Spontaneous bleeding rarely occurs, but bleeding can occur after surgery, which is more commonly seen in those with the lowest levels. The levels in this range, less than about 15 U/dL, constitute a major deficiency. Patients with partial deficiency have levels of about 20-60 U/dL (i.e., the lower limit of the normal range). About 30-50% of individuals with partial deficiency may still have excessive bleeding; however, identifying these persons in advance is difficult.

## Conclusions

In addition to thrombocytopenia, coagulopathy also occurs in dengue fever, which can precipitate the underlying pre-existing coagulopathy due to factor deficiency. Individuals who have haemophilia may have bleeding as early as the initial stage of fever and have greater platelet counts than most of the dengue patients. Therefore, a treating physician must remember that a patient presenting with persistent bleeding in a diagnosed dengue fever with rising platelet counts should be evaluated for other causes of bleeding promptly, henceforth reducing the mortality and prolonged hospitalisation.

## References

[1] Wijayaratne D, Ranasinghe P, Mohotti SP, Dilrukshi SA, Katulanda P (2015). Dengue fever in a patient with severe haemophilia: a case report. BMC Res Notes.

[2] Na-Nakorn S, Suingdumrong A, Pootrakul S, Bhamarapravati N (1966). Bone-marrow studies in Thai haemorrhagic fever. Bull World Health Organ.

[3] Funahara Y, Ogawa K, Fujita N, Okuno Y (1987). Three possible triggers to induce thrombocytopenia in dengue virus infection. Southeast Asian J Trop Med Public Health.

[4] Malasit P (1987). Complement and dengue haemorrhagic fever/shock syndrome. Southeast Asian J Trop Med Public Health.

[5] Chuansumrit A, Chaiyaratana W (2014). Hemostatic derangement in dengue hemorrhagic fever. Thromb Res.

[6] Isarangkura PB, Pongpanich B, Pintadit P, Phanichyakarn P, Valyasevi A (1987). Hemostatic derangement in dengue haemorrhagic fever. Southeast Asian J Trop Med Public Health.

[7] Funahara Y, Sumarmo Sumarmo, Shirahata A, Setiabudy-Dharma R (1987). DHF characterized by acute type DIC with increased vascular permeability. Southeast Asian J Trop Med Public Health.

[8] Chuansumrit A, Tangnararatchakit K, Sirachainan N (2012). Dengue infection in hematologic-oncologic pediatric patients: aggravation of anemia and bleeding risk. Southeast Asian J Trop Med Public Health.

[9] Brenner B, Laor A, Lupo H, Zivelin A, Lanir N, Seligsohn U (1997). Bleeding predictors in factor-XI-deficient patients. Blood Coagul Fibrinolysis.

